# Patients’ Perspectives and Challenges with Sacral Neuromodulation Implantable Pulse Generator

**DOI:** 10.1007/s00192-026-06756-8

**Published:** 2026-06-09

**Authors:** Mazen Mansour, Alexander Fahmy, Maria D’Amico, Mohammad El Hussein, Tyler Trump, Mahmoud Abou Zeinab, Ahmad Albakr, Howard B. Goldman

**Affiliations:** 1 Cleveland Clinic Foundation Glickman Urologic and Kidney Institute, Cleveland, OH USA; 2 Drexel University College of Medicine, Philadelphia, PA USA

**Keywords:** Sacral neuromodulation, Implantable pulse generator, Overactive bladder, Urinary incontinence, Patient-reported outcomes, Body mass index, Device tolerability

## Abstract

**Introduction:**

Sacral neuromodulation (SNM) is an established therapy for refractory bowel and lower urinary tract dysfunction. While its efficacy and patient satisfaction are well documented, little is known about the physical burden of the implant itself. This study aimed to evaluate patient perspectives on the implantable pulse generator (IPG) placed in the gluteal region.

**Methods:**

We reviewed 100 consecutive patients who underwent SNM within the past 2 years at a tertiary care center. Data were collected during follow-up visits or phone interviews using a five-question survey based on common patient concerns. Responses were analyzed descriptively, with statistical testing performed using SPSS version 29.

**Results:**

Eighty-two patients received SNM for overactive bladder, 16 for urinary retention, and two for fecal incontinence. Sixty-two percent reported awareness of the IPG, 18% reported sleep or movement limitations, and 36% reported activity-related discomfort. Eleven percent turned off the device for non-MRI reasons. Overweight or obese patients (BMI >25) were less likely to report disturbances compared with normal-weight patients. Male patients were more likely than female patients to deactivate their device (*P* = 0.014), while older patients were less likely to turn it off.

**Discussion:**

Most patients tolerated SNM well, though lower BMI was associated with greater discomfort, consistent with prior reports of higher revision rates in leaner individuals. Sex- and age-related differences suggest variations in device use behaviors, possibly due to lifestyle, activity level, or technological engagement.

**Conclusion:**

SNM is generally well tolerated, but leaner, younger, and male patients may experience greater device-related burden, highlighting the need for tailored counseling and device optimization.

## Introduction

Sacral neuromodulation (SNM) has emerged as a critical therapeutic option in the management of refractory bowel and lower urinary tract dysfunction, particularly when conservative and pharmacologic treatments fail. Fecal incontinence (FI), urinary frequency, urinary urgency incontinence (UUI), and nonobstructive urinary retention are highly prevalent and often debilitating. Overactive bladder (OAB), for instance, affects approximately 16% of adults in the United States, with a higher prevalence among women and older individuals [1]. Similarly, fecal incontinence is estimated to affect up to 8% of non-institutionalized adults, with incidence rising with age and parity [2].

SNM, commonly performed using the InterStim™ system (Medtronic), involves the implantation of a programmable pulse generator in the gluteal region to modulate sacral nerve roots, typically at the S3 foramen. The Medtronic system has been the most widely studied and extensively utilized neuromodulation platform since its FDA approval for urinary indications in 1997 and for fecal incontinence in 2011. This therapy has demonstrated efficacy in improving symptoms of urinary urgency incontinence, fecal incontinence, and nonobstructive urinary retention, with randomized controlled trials showing symptom improvement or resolution in 60–90% of patients [3, 4]. The InterStim device offers strengths, including reversibility, durability, and a strong evidence base across diverse patient populations. These advantages, combined with Medtronics widespread clinical adoption, make it a central focus when studying patient perspectives of implantable neuromodulation technology.

Other available devices and neuromodulation modalities include the Axonics Therapy™ system (Axonics Inc.), and posterior tibial nerve stimulation (PTNS), a less invasive but typically less durable alternative that requires ongoing office visits [4]. Overall, SNM provides a minimally invasive, reversible, and effective long-term solution with high rates of patient satisfaction and improvement in quality-of-life [5].

Despite the growing evidence supporting SNM in terms of clinical outcomes and patient satisfaction, there remains limited research examining the lived experience of patients with the implant, particularly with respect to physical perceptions of the device, discomfort, interference with daily activities, or aesthetic concerns. Understanding the physical burden and psychosocial impact of a gluteal implant may further inform shared decision-making, device design, and long-term adherence.

The aim of our study was to examine patients’ perspectives on the physical device and perceived burden of the implantable device in the gluteal region.

## Materials and Methods

One hundred consecutive patients who underwent Medtronic non-rechargeable SNM in the past 2 years were included in this study (Table 1). Institutional review board approval was obtained from Cleveland Clinic Foundation prior to initiation of the study (IRB 23–381). Procedures were performed at a large, tertiary academic center (The Cleveland Clinic Glickman Urological Institute). Patients included in the study did not receive any special preoperative counseling beyond our standard. Data was then collected either during a follow-up visit or a phone call at least 6 months after the procedure with a questionnaire based on five common patient questions (Table 2). The questionnaire was developed by fellowship-trained URPS (urogynecology and reconstructive pelvic surgery) surgeons with extensive experience with SNM. Responses were analyzed using descriptive statistics, and univariate analysis was performed using SPSS version 29. Subgroup analysis looking at patients grouped by BMI and sex were performed using chi-squared analysis (Table 3).

**Table 1:** Baseline characteristics of patients with IPG (*N* = 100)

Characteristic	Value
Age, years	
Mean (SD)	60.0 (18.0)
Non-elderly (< 65 years), *n* (%)	57 (57.0%)
Elderly (≥ 65 years), *n* (%)	43 (43.0%)
Gender, **n** (%)	
Female	93 (93.0%)
Male	7 (7.0%)
BMI, kg/m^2^	
Mean (SD)	29.0 (8.1)
Normal (< 24.9), *n* (%)	35 (35.0%)
Overweight (25–29.9), *n* (%)	26 (26.0%)
Obese (≥ 30), *n* (%)	39 (39.0%)
Indication for IPG, ***n*** (%)	
Overactive bladder	82 (82.0%)
Urinary retention	16 (16.0%)
Fecal incontinence	2 (2.0%)

**Table 2:** Patient questionnaire responses regarding IPG device

Question	Response (*n*, %)
**1. Do you notice/feel the location of your device (IPG)?**	
Yes	62 (62.0%)
No	38 (38.0%)
**2. Does the device limit your movements/sleeping positions at night?**	
Yes	18 (18.0%)
No	82 (82.0%)
**3. Does the device bother you while driving, cycling, or swimming?**	
Yes	36 (36.0%)
No	64 (64.0%)
**4. Do you turn off your device while doing certain activities**	
* (excluding MRI)?*	
Yes	11 (11.0%)
No	89 (89.0%)
**5. Do you get asked about or notice changes in stimulation when passing through airport security detectors? (*N* = 80)**	
Yes	16 (20.0%)
No	64 (80.0%)

**Table 3:** Common predictors

Questionnaire	Predictor	*P* value
Feel the presence of the IPG	None	> 0.05
Limit movement/sleep position	BMI **<**25 kg/m^2^	**0.001**
Discomfort from the IPG	None	> 0.05
Need to turn off the IPG	Younger age <65 years	**0.038**
Need to turn off the IPG	Gender Male	**0.014**
Airport security	None	>0.05

## Results

A total of 100 patients with an IPG for SNM were included in the study. The majority of patients were female (93%) with a mean age of 60.0 years (SD 18.0), and 43% were ≥65 years. The mean BMI was 29.0 kg/m^2^ (SD 8.1), with 35% of patients having a normal BMI (<24.9 kg/m^2^), 26% classified as overweight (25–29.9 kg/m^2^), and 39% classified as obese (≥30 kg/m^2^).

In terms of indication for SNM, 82 patients (82%) underwent implantation for OAB, 16 patients (16%) for urinary retention, and two patients (2%) for fecal incontinence (Table 1).

Regarding patient-reported outcomes, 62 patients (62%) reported awareness of the IPG’s physical location in the gluteal region, 18% indicated that the device limited their sleeping positions or physical movements at night, and 36% experienced some level of bother during activities such as driving, cycling, or swimming. Eleven percent of patients reported turning off their device for reasons other than undergoing MRI, including sleep, biking, driving, and swimming.

Among a subset of 80 patients who had traveled through airport security, 20% (*n* = 16) noted that they were either questioned about the device or noticed changes in stimulation during metal detector or scanner passage.

Subgroup analyses revealed patients who were overweight (BMI 25–29.9 kg/m^2^) or obese (BMI > 30 kg/m^2^), were less likely (*P* =.001) to report sleep or activity disturbances compared to patients with normal BMI (<24.9 kg/m^2^). In contrast, no statistically significant difference was found between overweight individuals and those with normal BMI in sensitivity to the physical presence of the IPG (*P* >.05). Although the overall male representation in the cohort was small (*n* = 7), men were significantly more likely to report turning off their device, due to physical discomfort, such as jolts, tingling, and uncomfortable stimulation during activities unrelated to MRI compared to women (*P* = 0.014). Additionally, age appeared to be inversely correlated with device deactivation behavior—older patients were less likely to turn off the device.

## Discussion

This study offers important insights into the physical experiences and perceived burden associated with SNM using an IPG. Although SNM has well-documented efficacy for lower urinary tract symptoms and fecal incontinence, limited research has explored patients’ day-to-day interactions with the implanted device itself, particularly in relation to physical comfort, lifestyle modifications, and behavioral adaptation.

In our cohort, 62% of patients reported perceiving the presence of the IPG. While this finding highlights the device’s detectability, the majority of patients did not experience meaningful disruption: only 18% reported interference with sleep or movement, and 36% noted activity-related discomfort. These figures align with previous literature showing that although patients may feel the implant, tolerability remains high and satisfaction with therapy is generally maintained over time [6, 7]. BMI appeared to influence patient-reported discomfort. Patients with a BMI >25 kg/m^2^ (classified as overweight or obese) were significantly less likely to report device-related disturbances. Despite the small sample size, this observation supports earlier findings that individuals with higher adiposity may experience less IPG prominence or irritation due to greater subcutaneous tissue coverage [8, 9]. Conversely, low BMI has been associated with higher revision rates due to discomfort, limitations in sleep position or IPG erosion, underscoring the importance of individualized patient selection and device placement planning [10].

Notably, a small proportion of patients (11%) reported voluntarily turning off the device during activities other than MRI. Interestingly, this behavior was more common in male patients (*P* = 0.014), though the sample size of men in the cohort was small (*n* = 7). The reason for this sex difference remains unclear but may reflect differences in activity type, tolerance of stimulation-related sensations, or approach to device management, rather than the physical presence of the IPG itself. Prior studies have suggested that men may have higher thresholds for tolerating stimulation-related sensations but may also be more inclined to modify device use during physical exertion [11].

Age was inversely correlated with the likelihood of deactivating the device, suggesting that older patients may be more accepting of the physical presence of the IPG or engage in less activity that exacerbates discomfort from the implant itself. This finding mirrors prior studies indicating that older adults are often more compliant with SNM therapy and report higher satisfaction, possibly due to more stable lifestyle patterns and lower physical demands. Additionally, older patients may be less inclined to interact frequently with the technological aspects of InterStim devices, whether due to lower perceived need, reduced familiarity or comfort with device programming, or a preference for minimal technological engagement—which could further contribute to their lower rates of device deactivation [12]. Moreover, broader research on digital medical services suggests that older adults may experience higher perceived risk and lower behavioral intention regarding new technology, reinforcing the notion that they are less likely to engage proactively with device-related technology [13].

Regarding device interactions in public settings, only 20% of patients who traveled reported being questioned about their device or experiencing detectable stimulation changes during airport security screening. This low incidence supports current guidance that modern SNM systems are largely unaffected by routine security measures, although it underscores the importance of providing patients with device identification cards and education on navigating such scenarios. Medtronics patient guidance similarly advises carrying an identification card, informing security personnel, and, if necessary, temporarily turning off the device before screening. Practices that align with these findings and help ensure a smoother travel experience [13, 14].

Taken together, these findings support the favorable tolerability profile of SNM, and the minimal day-to-day burden experienced by most recipients. However, certain subgroups, particularly those with low BMI, may benefit from closer counseling about implant sensation and site discomfort, or surgeons may take extra care to ensure the IPG is implanted deep enough in an adequate location. Device design innovations, including smaller or rechargeable IPGs, may further reduce the burden in these populations [14].

This study has several limitations. It was conducted at a single tertiary care center, which may limit the generalizability of the findings to other patient populations or practice settings. The fact that we did not have a validated questionnaire also diminishes the generalizability of our study findings, but it is noteworthy that there is no validated questionnaire in the literature on the subject matter. The data was collected through patient self-report during follow-up visits or phone calls, introducing the potential for recall bias and social desirability bias. The study cohort was predominantly female (93%), limiting the ability to draw conclusions about sex-specific differences. There were a very small number of men in the cohort (7); therefore, it is difficult to draw any conclusions about this group, but our results may be hypothesis-generating for future studies. The cross-sectional design precludes assessment of how device-related perceptions or burdens may change over time. Despite these limitations, the notable strengths of the study include that all patients were treated with Medtronic InterStim non-rechargeable devices, which ensures consistency in device type and allows the findings to be directly linked to the most widely used and well-validated neuromodulation system. Second, the large sample size of 100 patients provides meaningful insight into real-world patient experiences across a range of demographics and clinical indications. Finally, the questionnaire, although not validated, was based on patient-centered questions that reflect real concerns commonly raised during counseling—providing practical and clinically relevant insights for providers.

## Conclusion

In this study, we found that while a majority of patients are aware of their sacral neuromodulation implant, the physical and functional burden of the device is generally low. Most patients tolerate the implant well, with minimal disruption to sleep, activity, or daily routines. Importantly, individual factors such as BMI, age, and sex may influence patients’ experiences, suggesting a need for personalized preoperative counseling and postoperative support. While our small sample size limits the ability to make robust conclusions, our findings can serve as hypothesis-generating for future inquiries and highlight the need for larger studies.

As sacral neuromodulation technology continues to evolve, future research should focus on optimizing device design—such as miniaturization, optimizing device depth while still allowing comfort and optimal function, or alternative anatomical sites—to further reduce implant-related discomfort, especially in leaner patients. Longitudinal studies exploring the psychosocial impact, long-term tolerability, and quality-of-life outcomes across diverse patient populations will also be essential for refining patient selection and improving therapy adherence.
